# Supplementary material for: HIVprotI: an integrated web based platform for prediction and design of HIV proteins inhibitors
Source: J Cheminform. 2018 Mar 9;10:12. doi: 10.1186/s13321-018-0266-y (PMC5845081; doi:10.1186/s13321-018-0266-y)
Supplement: Supplementary file 2 — Additional file 2. Source code of HIVProtI web server. [file 13321_2018_266_MOESM2_ESM.zip › HIVprotI_Source-code/hivprotI/weka-3-6-6/doc/weka/experiment/OutputZipper.html]

OutputZipper


---


|  |  |  |
| --- | --- | --- |
| **Overview** \| **Package** \| **Class** \| **Tree** \| **Deprecated** \| **Index** \| **Help** \| **Weka's home** | |  |
| **PREV CLASS**   **NEXT CLASS** | **FRAMES**    **NO FRAMES**     **All Classes** |  |
| SUMMARY: NESTED \| FIELD \| CONSTR \| METHOD | DETAIL: FIELD \| CONSTR \| METHOD |  |


---


## weka.experiment Class OutputZipper

```
java.lang.Object
  weka.experiment.OutputZipper
```

**All Implemented Interfaces:**: RevisionHandler

---

``` public class OutputZipper extends java.lang.Object implements RevisionHandler ```

OutputZipper writes output to either gzipped files or to a
multi entry zip file. If the destination file is a directory
each output string will be written to an individually named
gzip file. If the destination file is a file, then each
output string is appended as a named entry to the zip file until
finished() is called to close the file.

**Version:**
:   $Revision: 1.8 $

**Author:**
:   Mark Hall (mhall@cs.waikato.ac.nz)

---

**Constructor Summary**

`OutputZipper(java.io.File destination)`
  
          Constructor.


| **Method Summary** | |
| --- | --- |
| `void` | `finished()`             Closes the zip file. |
| `java.lang.String` | `getRevision()`             Returns the revision string. |
| `static void` | `main(java.lang.String[] args)`             Main method for testing this class |
| `void` | `zipit(java.lang.String outString, java.lang.String name)`             Saves a string to either an individual gzipped file or as an entry in a zip file. |

**Methods inherited from class java.lang.Object**

`equals, getClass, hashCode, notify, notifyAll, toString, wait, wait, wait`

**Constructor Detail**

### OutputZipper

```
public OutputZipper(java.io.File destination)
             throws java.lang.Exception
```

:   Constructor.

    **Parameters:**: `destination` - a destination file or directory **Throws:**: `java.lang.Exception` - if something goes wrong.


**Method Detail**

### zipit

```
public void zipit(java.lang.String outString,
                  java.lang.String name)
           throws java.lang.Exception
```

:   Saves a string to either an individual gzipped file or as
    an entry in a zip file.

    :   **Parameters:**: `outString` - the output string to save: `name` - the name of the file/entry to save it to **Throws:**: `java.lang.Exception` - if something goes wrong

---


### finished

```
public void finished()
              throws java.lang.Exception
```

:   Closes the zip file.

    :   **Throws:**: `java.lang.Exception` - if something goes wrong

---


### getRevision

```
public java.lang.String getRevision()
```

:   Returns the revision string.

    :   **Specified by:**: `getRevision` in interface `RevisionHandler`
    :   **Returns:**: the revision

---


### main

```
public static void main(java.lang.String[] args)
```

:   Main method for testing this class


---


|  |  |  |
| --- | --- | --- |
| **Overview** \| **Package** \| **Class** \| **Tree** \| **Deprecated** \| **Index** \| **Help** \| **Weka's home** | |  |
| **PREV CLASS**   **NEXT CLASS** | **FRAMES**    **NO FRAMES**     **All Classes** |  |
| SUMMARY: NESTED \| FIELD \| CONSTR \| METHOD | DETAIL: FIELD \| CONSTR \| METHOD |  |


---
